# Association Between Blood Pressure Variability and Acute Kidney Injury Following Transcatheter Aortic Valve Replacement: A Study Based on the Medical Information Mart for Intensive Care-IV Database

**DOI:** 10.1093/icvts/ivag227

**Published:** 2026-08-14

**Authors:** Ruowen Li, Zhao Li, Bo Zhang

**Affiliations:** Department of Anesthesiology, China-Japan Friendship Hospital, Beijing 100029, China; Department of Anesthesiology, China-Japan Friendship Hospital, Beijing 100029, China; Department of Anesthesiology, China-Japan Friendship Hospital, Beijing 100029, China

**Keywords:** blood pressure variability, transcatheter aortic valve replacement, acute kidney injury

## Abstract

**Objectives:**

Acute kidney injury (AKI) following transcatheter aortic valve replacement (TAVR) is a prevalent complication that impacts patient prognosis. While blood pressure variability (BPV) has been implicated in AKI among patients undergoing other surgical procedures, its association with AKI following TAVR remains uncharted. This study aimed to investigate the association between BPV and post-TAVR AKI.

**Methods:**

Based on the Medical Information Mart for Intensive Care database, logistic regression models were employed to investigate the associations of average real variability of systolic blood pressure (ARV-SBP) and average real variability of diastolic blood pressure (ARV-DBP) with AKI following TAVR. Multiple sensitivity analyses evaluated the robustness of the results. The marginal standardization analysis estimated the odds of AKI after ARV-SBP adjustment.

**Results:**

A total of 340 patients undergoing TAVR were enrolled, among whom 107 (31.5%) developed AKI. Logistic regression analysis demonstrated a positive correlation between ARV-SBP and the odds of post-TAVR AKI (odds ratio [OR] = 1.34, 95% confidence interval [CI]: 1.03-1.74, *P *= .029) in the fully adjusted model. In contrast, no significant correlation was observed between ARV-DBP and AKI (*P *= .559). In covariate and blood pressure measurement sensitivity analyses, the direction of the ARV-SBP effect was generally consistent with that in the primary analysis. Marginal standardization analysis demonstrated that higher ARV-SBP levels were associated with elevated odds of AKI.

**Conclusions:**

Elevated ARV-SBP was associated with increased odds of post-TAVR AKI. Average real variability of systolic blood pressure may serve as an auxiliary indicator for early risk identification of post-TAVR AKI, yet its clinical value remains to be validated in prospective studies.

## Graphical abstract

**Figure:**
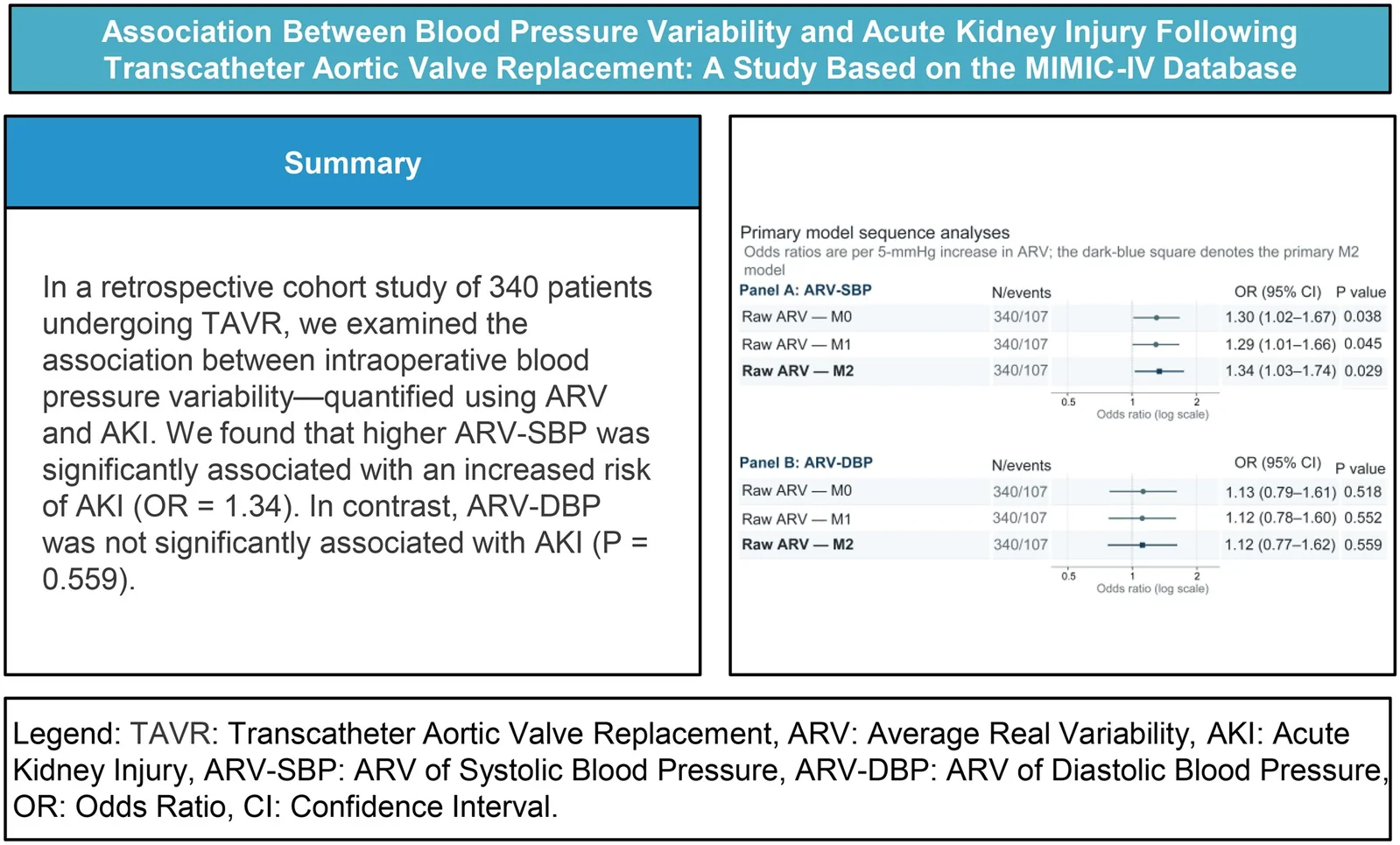

## INTRODUCTION

Transcatheter aortic valve replacement (TAVR) has emerged as the first-line treatment for patients with severe aortic stenosis who are at high surgical risk or have surgical contraindications.^1^ With continuous advancements in device technology and operation pathway, the success rate of TAVR has exceeded 95%.^2^^,^^3^ Despite the increasing maturity of the technology, the prophylaxis and treatment of perioperative complications remain a critical clinical priority. Acute kidney injury (AKI) ranks among the prevalent and severe complications following TAVR. Data from the American College of Cardiology National Cardiovascular Data Registry exhibit that among 107 814 patients with TAVR, 11 566 (10.7%) developed postoperative AKI.^4^ Furthermore, AKI following TAVR is associated with elevated mortality and prolonged hospital stays. A meta-analysis reveals that the 30-day mortality rate among TAVR patients with AKI ranges from 7.8% to 29.0%, representing an increase of 2- to 8-fold compared to patients without AKI, accompanied by an increase of 2.5-fold prolongation of hospital stays.^5^ More severely, progression to stage 3 AKI is associated with a 7-fold higher 1-year mortality rate in patients with TAVR compared to those without AKI.^4^ Thus, identifying determinants associated with AKI following TAVR progression and implementing targeted management are crucial for improving patient clinical outcomes.

Among acute interventional procedures, patients with TAVR experience frequent blood pressure variability (BPV) secondary to haemodynamic perturbations induced by anaesthesia induction, rapid ventricular pacing, and valve deployment.^6^^,^^7^ Notably, BPV, as a metric capturing blood pressure changes,^8^ has increasingly been implicated in the postoperative AKI among surgical patients. A retrospective analysis of 369 patients undergoing elective surgery demonstrates that elevated intraoperative systolic blood pressure (SBP) and diastolic blood pressure (DBP) variability are independently associated with an increased risk of AKI.^9^ Within the field of cardiac surgery, a study targeting patients who undergo coronary artery bypass grafting further confirmed that elevated SBP variability is an independent risk factor for postoperative renal failure (odds ratio [OR] = 1.14).^10^ Meanwhile, in paediatric cardiac surgery, intraoperative SBP and DBP variability were both associated with an elevated incidence of AKI in patients undergoing cardiopulmonary bypass surgery (both OR = 1.40).^11^ Collectively, we hypothesize that BPV may also be related to the pathogenesis and progression of AKI following TAVR.

Thus, based on the Medical Information Mart for Intensive Care (MIMIC) database, the association between blood pressure (SBP and DBP) variability and AKI following TAVR is investigated. The aim is to provide a scientific basis for the management of AKI following TAVR.

## METHODS

### Study population

The data were derived from the MIMIC-IV (version 2.2) database, which was jointly maintained by Beth Israel Deaconess Medical Center and the Massachusetts Institute of Technology. This database encompassed medical records of patients admitted to the intensive care unit (ICU) between 2008 and 2019. Given that all patient information within the database was fully anonymized, no additional ethical approval was required.

A total of 760 patients undergoing TAVR were initially identified in this study. The exclusion criteria were as follows: (1) patients who were not admitted to the ICU on the day of the procedure or the subsequent day (*n* = 306); (2) patients with an ICU stay shorter than 24 hours (*n* = 34); (3) patients with an insufficient number of valid paired blood pressure recordings or an inadequate time span of blood pressure monitoring (*n* = 36); (4) patients who met the diagnostic criteria for AKI based on serial creatinine testing prior to the index procedure date (*n* = 20); (5) patients with missing covariates required for model 2 (*n* = 24). Ultimately, 340 eligible participants were included in the final cohort, among whom 107 developed AKI (**Figure 1**).

**Figure 1.**
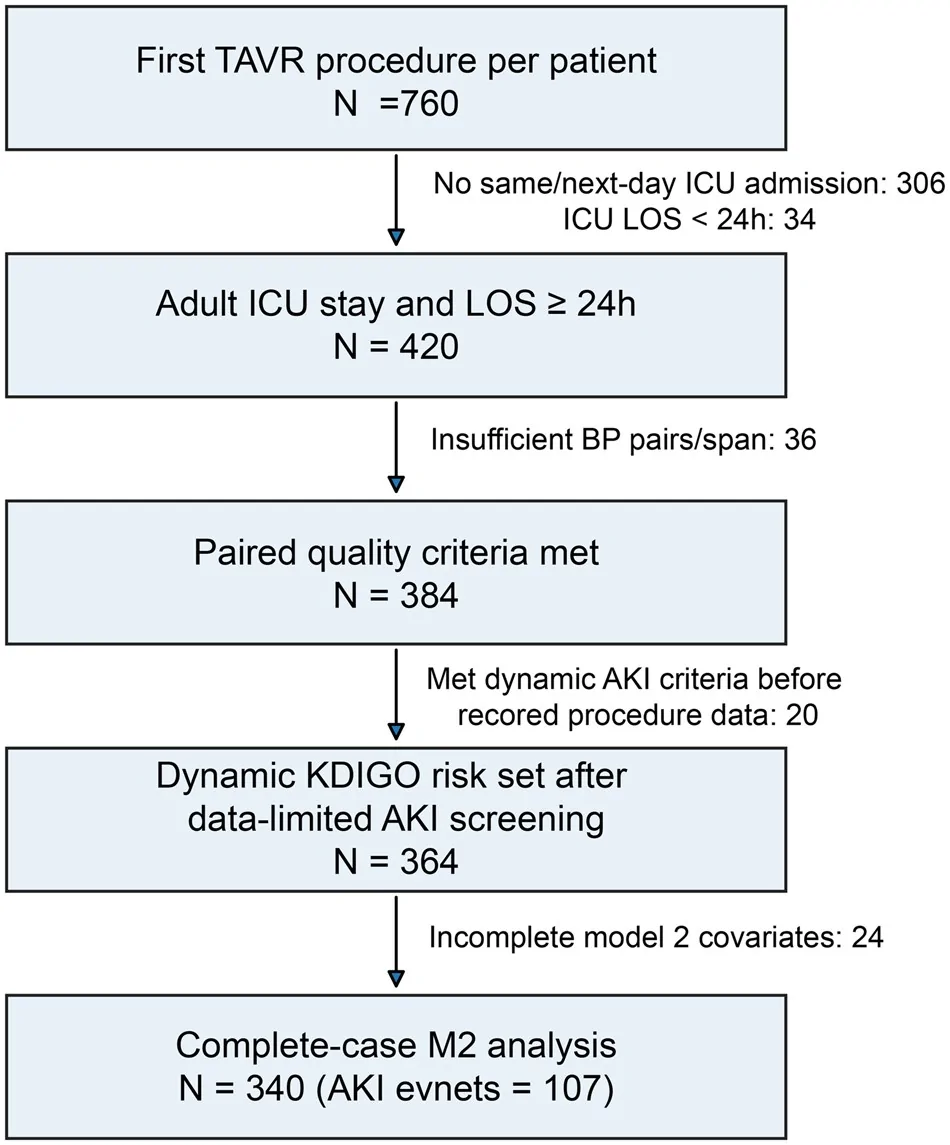
Inclusion and Exclusion of Participants. Abbreviations: AKI, acute kidney injury; BP, blood pressure; ICU, intensive care unit; KDIGO, Kidney Disease: Improving Global Outcomes; LOS, length of stay; TAVR, transcatheter aortic valve replacement

### Definition of disease

Transcatheter aortic valve replacement diagnoses were identified using the International Classification of Diseases, 9th revision and 10th revision codes extracted from the “procedures_icd” table of the MIMIC-IV database; further details were provided in **Table S1**.

### Associated variables

#### Exposure variables

The primary exposures of this study were average real variability of systolic blood pressure (ARV-SBP) and average real variability of diastolic blood pressure (ARV-DBP) within the 24-hour window after ICU admission, defined as [ICU intime, ICU intime + 24 h). Invasive blood pressure values were extracted using itemid 220050 (invasive arterial systolic blood pressure) and 220051 (invasive arterial diastolic blood pressure). Non-invasive blood pressure values were retrieved via itemid 220179 (non-invasive systolic blood pressure) and 220180 (non-invasive diastolic blood pressure). For duplicate measurements recorded at the identical charttime and from the same blood pressure source, the median values of SBP and DBP were calculated separately. Any blood pressure records meeting any of the following criteria were excluded: warning = 1; SBP outside the range of 40-300 mmHg; DBP outside the range of 20-200 mmHg; missing SBP or DBP values; SBP ≤ DBP; or pulse pressure (SBP − DBP) outside the range of 10-200 mmHg. When both valid invasive and non-invasive blood pressure pairs were available at the same charttime, invasive measurements were prioritized.

All quality-controlled blood pressure pairs were sorted chronologically by charttime. The raw ARV was defined as the arithmetic mean of absolute differences between 2 consecutive valid blood pressure measurements:


$$\mathrm{ARV}=\frac{1}{m-1}\sum_{j=2}^{m}\left|{BP}_{j}-{BP}_{j-1}\right|$$

*m* denotes the number of valid blood pressure measurement pairs, and *BP_j_* represents the value of the *j*-th measurement. For the primary analysis, each patient was required to have a minimum of 6 valid blood pressure pairs, with a time span of at least 12 hours between the first and last recordings.

#### Outcome variables

The primary outcome of this study was KDIGO (Kidney Disease: Improving Global Outcomes) creatinine-only AKI occurring within 7 days after ICU admission among patients without preprocedural creatinine-detected AKI identified via serial creatinine testing prior to the index procedure date. At each evaluable serum creatinine measurement time point, the current creatinine value was compared with the minimum creatinine value within the preceding 48 hours and the minimum value within the preceding 7 days, respectively.

Acute kidney injury staging was performed as follows. Stage 1 was defined as an increase in serum creatinine of ≥0.3 mg/dL relative to the minimum value in the prior 48 hours, or a creatinine level ≥1.5 times the minimum value in the preceding 7 days. Stage 2 was defined as a creatinine level ≥2.0 times the minimum value within the prior 7 days. Stage 3 was defined as a creatinine level ≥3.0 times the minimum value within the preceding 7 days, or a serum creatinine concentration ≥4.0 mg/dL accompanied by either an absolute increase of ≥0.3 mg/dL from the 48-hour lowest value or a ≥ 1.5-fold rise relative to the 7-day lowest value. The peak creatinine stage within 7 days following ICU admission was recorded as the final patient-level stage. A peak stage of ≥1 was defined as the primary binary AKI outcome.

To identify pre-existing creatinine-based AKI prior to the index procedure date, identical serial creatinine diagnostic criteria used for the primary outcome were applied to serial creatinine measurements collected within the 7 days before the procedure date. For each measurement, the current creatinine level was compared separately with its 48-hour lowest value and 7-day lowest value. Patients with any measurement meeting KDIGO stage 1 or higher were classified as having preprocedural AKI and excluded from the primary risk set. Since procedures_icd.chartdate only records calendar dates without exact procedural timestamps, this screening could not capture AKI developing from 00:00 on the index procedure date until the actual operation or ICU admission. Therefore, this approach did not enable precise identification of true preoperative AKI.

A secondary composite KDIGO outcome integrating serum creatinine, urine output, and continuous renal replacement therapy (CRRT) criteria was additionally defined. At each evaluable time point, separate stages were assigned based on creatinine, urine output, and CRRT criteria; any valid CRRT record was categorized as KDIGO stage 3. The highest stage across the 3 metrics was defined as the composite KDIGO stage at that time point. The peak composite stage within 7 days after ICU admission represented the patient-level secondary outcome, and a peak composite stage ≥1 was defined as composite KDIGO AKI.

#### Covariates

Major covariates were predefined prior to analyses based on clinical knowledge and a prespecified directed acyclic graph (DAG) (**Figure S1**).

Intravenous fluid intake, fluid output, fluid balance, diuretic administration, vasopressor use, epinephrine exposure, and invasive mechanical ventilation recorded within the initial 24 hours after ICU admission overlapped temporally with the ARV exposure window (**Table S2**). These variables may reflect concurrent illness severity or lie along potential causal pathways between ARV and AKI. Accordingly, they were only described as early postoperative management characteristics and not included in the primary fully adjusted model 2.

### Statistical analysis

Structured Query Language was employed to extract data from the MIMIC-IV (version 2.2) database.

Continuous variables were summarized according to their distribution characteristics: mean (standard deviation) was reported when the absolute value of sample skewness was <1; otherwise, median [interquartile range] was presented. Categorical variables were described as counts and percentages. For comparisons of continuous variables between the AKI and non-AKI groups, Welch’s *t*-test was used if the absolute skewness was <1, while the Mann-Whitney U test was applied for skewed distributions. Pearson’s chi-square test was adopted for categorical variables, with Fisher’s exact test used when expected cell frequencies were less than 5.

Logistic regression models were fitted to assess the associations of ARV-SBP and ARV-DBP with creatinine-only KDIGO AKI, separately. Model 0 (M0) was an unadjusted model containing only the corresponding ARV exposure. Model 1 (M1) additionally adjusted for age and sex on the basis of M0. Model 2 (M2), the fully adjusted model, further incorporated the median serum creatinine measured within 7 days prior to the index procedure date, diabetes mellitus, and congestive heart failure. To ensure comparability across models, M0, M1 and M2 were restricted to the identical complete case sample used for M2.

Sensitivity analyses were performed in 3 main parts: (1) 3 sensitivity models were established to test the robustness of findings against covariate selection. ① Extended baseline model 3 (M3): M2 plus hypertension, prior myocardial infarction, atrial fibrillation/flutter, and chronic pulmonary disease. ② Estimated glomerular filtration rate (eGFR) substitution model: the median preprocedural 7-day creatinine was replaced with eGFR calculated via the 2021 race-free CKD-EPI equation.^12^ ③ Charlson substitution model: diabetes mellitus and congestive heart failure were replaced with the Charlson Comorbidity Index. (2) Blood pressure measurement-related sensitivity analyses. (3) Sensitivity analyses using 5 alternative fixed reference creatinine values to verify robustness against reference creatinine definition: Model stability was evaluated by the event-to-parameter ratio, maximum variance inflation factor (VIF), and model convergence status; no substantial multicollinearity was detected (**Table S3**). Firth penalized logistic regression was implemented when the event-to-parameter ratio fell below 5, and this method was also adopted for the prespecified analysis of creatinine-only KDIGO stage 2-3 AKI.

Marginal standardization analysis was applied to estimate adjusted AKI odds at the 25th, 50th, and 75th percentiles of the ARV-SBP distribution. The 95% confidence intervals (CIs) for adjusted risks were derived from 1000 patient-level bootstrap resamples. Absolute risk differences between the 75th and 25th ARV-SBP percentiles and their corresponding 95% CIs were additionally estimated.

All statistical analyses and figure plotting were conducted using Python. All statistical tests were 2-tailed, and a *P* value <.05 was considered statistically significant.

## RESULTS

### Characteristics of the study population

A total of 340 patients who underwent TAVR were enrolled, with 107 cases (31.5%) developing AKI. Females constituted a slight majority among patients undergoing TAVR, with a median age of 85 years (**Table 1**). Compared with patients without AKI, those who developed AKI had a higher median serum creatinine within 7 days before the index procedure date (1.2 vs 1.0, *P *< .001) and a greater prevalence of prior kidney disease (50.5% vs 29.6%, *P *< .001). In addition, patients with postoperative AKI exhibited higher Charlson Comorbidity Index scores and elevated ARV-SBP values, alongside lower eGFR and lower 24-hour mean SBP and mean DBP (all *P *< .05) (**Table 1**).

**Table 1.** Characteristics of the Primary Complete-Case Cohort According to Dynamic Creatinine-Based KDIGO AKI Status

Characteristic	Overall (*N* = 340)	No AKI (*N* = 233)	AKI (*N* = 107)	*P* value
Baseline clinical characteristics				
Age, years	85 [78.8, 89]	85 [79, 89]	84 [78, 88.5]	.571
Median serum creatinine during the 7 days before the recorded procedure date, mg/dL	1.1 [0.9, 1.4]	1 [0.8, 1.3]	1.2 [0.97, 1.58]	<.001
eGFR based on the 7-day median creatinine, mL/min/1.73 m^2^	59.8 ± 21.4	63.2 ± 20.4	52.4 ± 21.7	<.001
Charlson Comorbidity Index	7.06 ± 1.97	6.73 ± 1.82	7.79 ± 2.09	<.001
Male sex	163 (47.9%)	115 (49.4%)	48 (44.9%)	.441
Renal disease	123 (36.2%)	69 (29.6%)	54 (50.5%)	<.001
Hypertension	290 (85.3%)	195 (83.7%)	95 (88.8%)	.218
Prior myocardial infarction	71 (20.9%)	45 (19.3%)	26 (24.3%)	.294
Atrial fibrillation/flutter	174 (51.2%)	118 (50.6%)	56 (52.3%)	.772
Chronic pulmonary disease	139 (40.9%)	90 (38.6%)	49 (45.8%)	.212
Diabetes mellitus	122 (35.9%)	75 (32.2%)	47 (43.9%)	.036
Congestive heart failure	265 (77.9%)	177 (76%)	88 (82.2%)	.195
Blood pressure and measurement characteristics during the first 24 hours				
Mean SBP, mmHg	126 ± 13.3	127 ± 12.9	123 ± 13.6	.007
Mean DBP, mmHg	50.5 ± 7.58	51.1 ± 7.46	49.2 ± 7.71	.035
ARV-SBP, mmHg	11.9 [9.57, 15]	11.4 [9.32, 14.7]	12.7 [10.7, 15.2]	.015
ARV-DBP, mmHg	5.58 [4.31, 7.46]	5.7 [4.41, 7.43]	5.4 [4.26, 8.52]	.813
Valid BP pairs	29 [25, 34]	28 [25, 34]	29 [25, 37.5]	.296
Median sampling interval, min	60 [54, 60]	60 [59, 60]	60 [30.5, 60]	.006
Valid observation hours	22 [21, 23]	22 [21, 23]	22 [20, 23]	.348
BP source				.498
Invasive only	124 (36.5%)	85 (36.5%)	39 (36.4%)	
Non-invasive only	21 (6.2%)	12 (5.2%)	9 (8.4%)	
Mixed	195 (57.4%)	136 (58.4%)	59 (55.1%)	

Values are presented as mean (SD), median [IQR], or *n* (%). Continuous variables were compared using Welch’s t test when the absolute sample skewness was <1 and the Mann-Whitney U test otherwise. Categorical variables were compared using Pearson’s chi-square test or Fisher’s exact test when expected cell counts were <5. The *P* value for BP source was obtained from an overall Pearson chi-square test across the 3 categories. All *P* values are 2-sided.

Abbreviations: AKI, acute kidney injury; ARV, average real variability; BP, blood pressure; DBP, diastolic blood pressure; eGFR, estimated glomerular filtration rate; KDIGO, Kidney Disease: Improving Global Outcomes; SBP, systolic blood pressure.

Patients who developed AKI received greater fluid infusion volumes, demonstrated a more positive fluid balance, and were administered diuretics, vasopressors, epinephrine, and invasive mechanical ventilation more frequently (**Table S2**).

### Association between ARV-SBP, ARV-DBP, and AKI following TAVR

#### Primary outcome analysis

In the fully adjusted model 2 (M2), ARV-SBP was positively associated with creatinine-only KDIGO AKI following TAVR (OR = 1.34, 95% CI, 1.03-1.74, *P *= .029). By contrast, no significant association was observed between ARV-DBP and creatinine-only KDIGO AKI (*P *= .559) (**Figure 2A**, **Table S4**).

**Figure 2.**
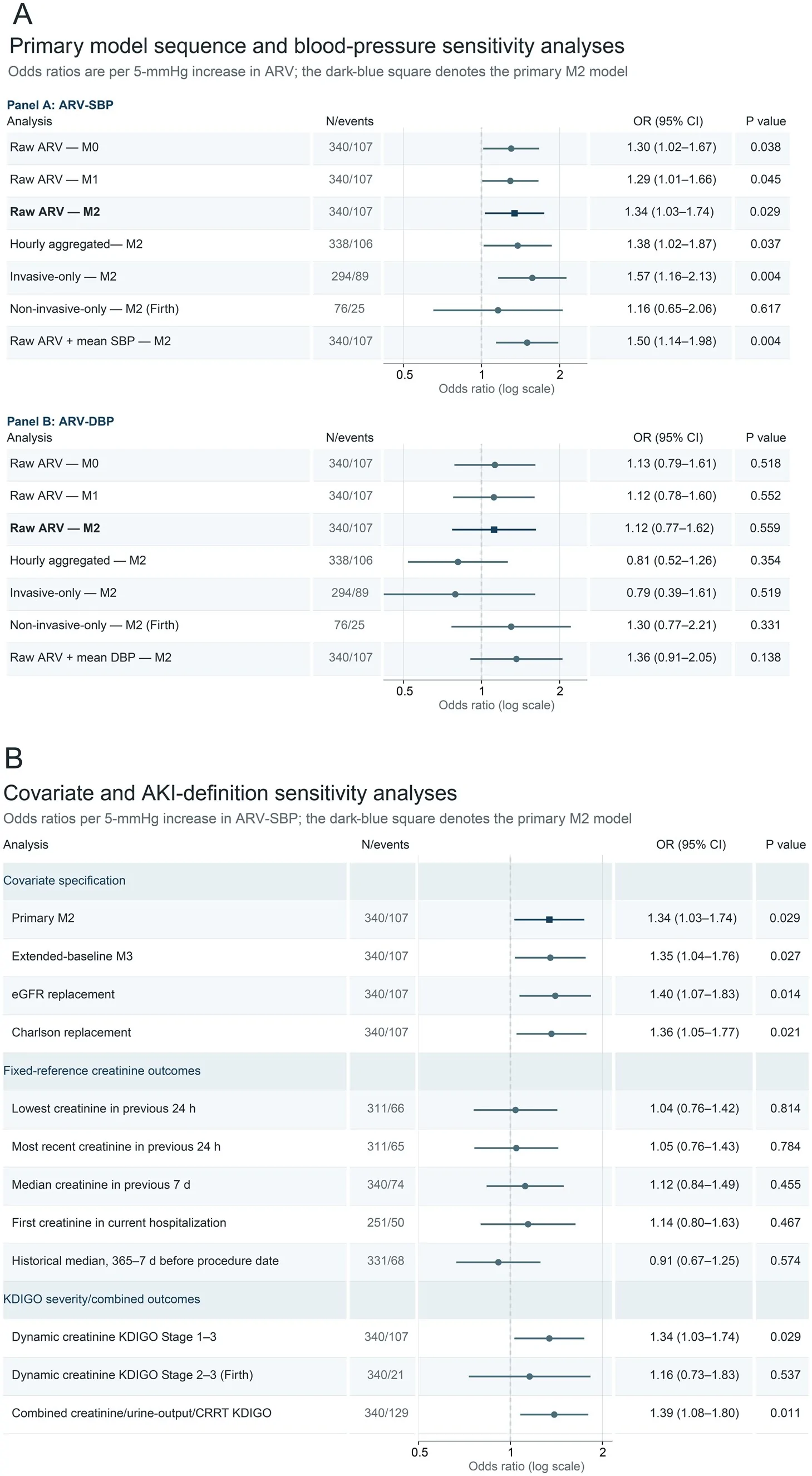
Associations of 24-Hour Blood Pressure Average Real Variability With Dynamic Creatinine-Based KDIGO Acute Kidney Injury and Sensitivity Analyses. (A) Associations of 24-hour ARV-SBP and ARV-DBP with dynamic creatinine-based KDIGO AKI across the primary model sequence and blood pressure measurement sensitivity analyses. Odds ratios are expressed per 5-mmHg increase in ARV. (B) Sensitivity analyses of the association between 24-hour ARV-SBP and AKI according to covariate specification and AKI outcome definition. ORs are expressed per 5-mmHg increase in ARV-SBP. The vertical dashed line indicates an OR of 1.0. The stage 2-3 analysis was performed using Firth penalized logistic regression, and the combined KDIGO outcome incorporated serum creatinine, urine output, and CRRT criteria. Exact 2-sided *P* values are shown in the rightmost column. Abbreviations: AKI, acute kidney injury; ARV-DBP, average real variability of diastolic blood pressure; ARV-SBP, average real variability of systolic blood pressure; CRRT, continuous renal replacement therapy; eGFR, estimated glomerular filtration rate; KDIGO, Kidney Disease: Improving Global Outcomes

#### Sensitivity analyses

Furthermore, ARV-SBP estimates were broadly consistent with those from Model M2 across covariate and blood pressure measurement sensitivity analyses (**Figure 2A and B**, **Table S5**). However, across sensitivity analyses utilizing the 5 fixed reference creatinine definitions to identify AKI, all effect estimates for ARV-SBP were close to the null value, and all corresponding 95% CIs included 1 (**Figure 2B**, **Table S6**).

By comparison, no consistent statistically meaningful association was detected between ARV-DBP and creatinine-only KDIGO AKI across all blood pressure measurement sensitivity analyses (all *P *> .05) (**Figure 2A**, **Table S5**).

#### KDIGO staging and secondary outcome analysis

Stratified analyses by AKI stage demonstrated that for creatinine-only KDIGO stage 1-3 AKI, each 5 mmHg increment in ARV-SBP was associated with an approximately 34% higher odds of AKI (OR = 1.34, 95% CI, 1.03-1.74, *P *= .029). When the outcome was restricted to more severe stage 2-3 AKI, no statistically significant association was detected (*P *= .537). This was likely driven by the limited number of only 21 events, leading to imprecise effect estimates from Firth penalized logistic regression (**Figure 2B**, **Tables S6 and S7**).

For the composite KDIGO secondary outcome incorporating serum creatinine, urine output, and CRRT criteria, the direction of the association for ARV-SBP remained consistent with the primary analysis (OR = 1.39, 95% CI, 1.08-1.80, *P *= .011) (**Figure 2B**, **Table S6**).

### Absolute risk

Marginal standardization based on Model M2 revealed that elevated 24-hour ARV-SBP was associated with a higher adjusted risk of creatinine-only KDIGO AKI. Model-estimated AKI probability rose progressively with increasing ARV-SBP, and the adjusted absolute risk difference between the 75th and 25th percentiles of ARV-SBP was 6.3 percentage points (95% CI, 0.2-12.2) (**Figure 3**). Of note, this curve constitutes a model-dependent descriptive estimate rather than a causal effect and cannot be used to define clinical intervention cutoffs.

**Figure 3.**
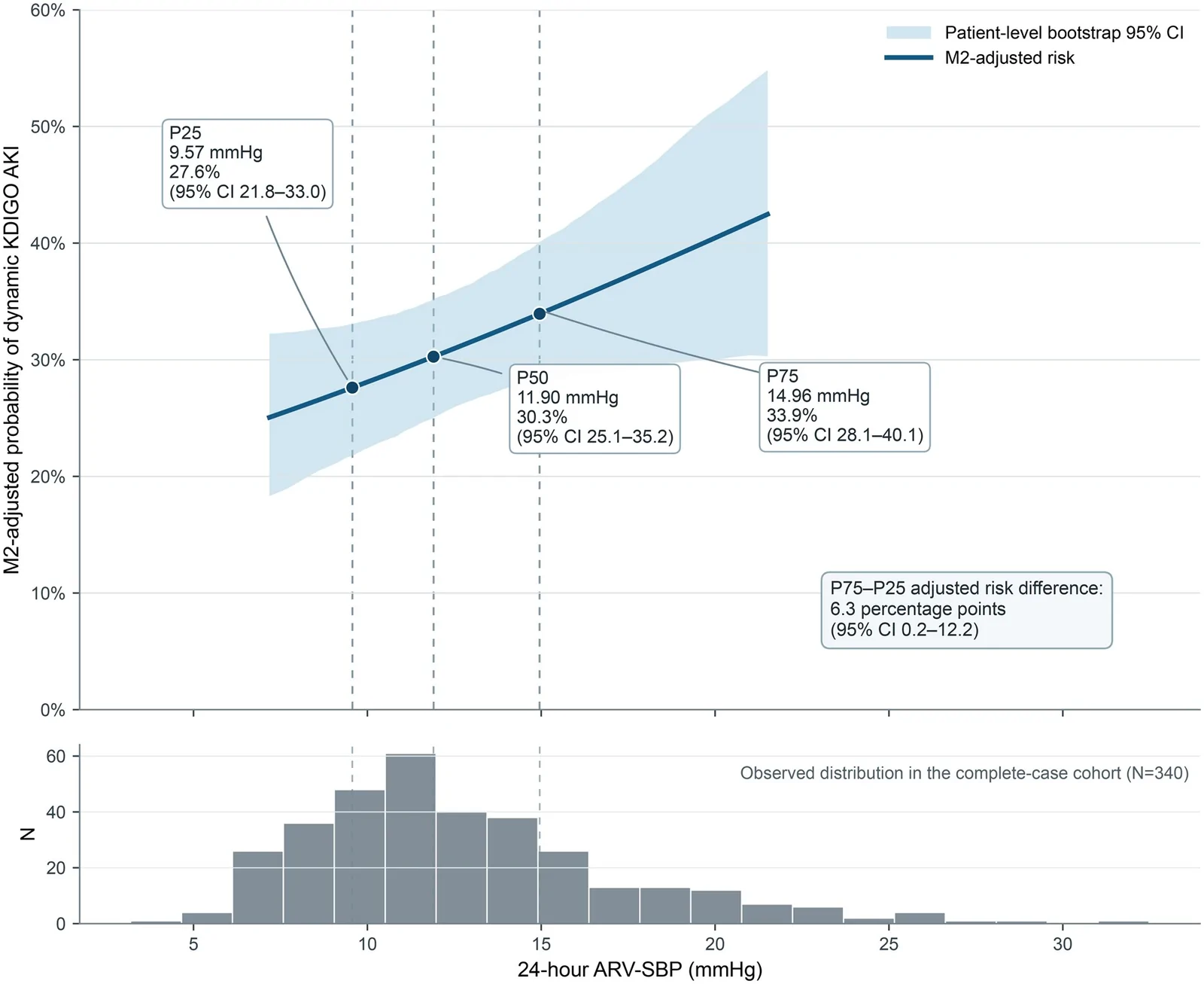
M2-Adjusted Probability of Dynamic Creatinine-Based KDIGO AKI Across Continuous 24-Hour ARV-SBP Values. The solid line represents the marginally standardized risk estimated from the primary M2 model, and the shaded area represents the patient-level bootstrap 95% confidence interval. Points and vertical dashed lines indicate the 25th, 50th, and 75th percentiles of ARV-SBP—9.57, 11.90, and 14.96 mmHg—corresponding to adjusted AKI risks of 27.6%, 30.3%, and 33.9%, respectively. The curve is a descriptive, model-based estimate and does not establish causality or define a clinical intervention threshold. Abbreviations: AKI, acute kidney injury; ARV-SBP, average real variability of systolic blood pressure; CI, confidence interval; KDIGO, Kidney Disease: Improving Global Outcomes

## DISCUSSION

Our study found that higher ARV-SBP was associated with elevated odds of AKI within 7 days after TAVR. This association maintained a consistent directional trend across covariate adjustment schemes and multiple blood pressure measurement sensitivity analyses, yet attenuated when AKI was defined using fixed reference creatinine values. No significant correlation was observed between ARV-DBP and postoperative AKI.

Average real variability of systolic blood pressure was positively correlated with AKI following TAVR, whereas ARV-DBP exhibited no association with postoperative AKI. The results were aligned with prior observations in other cardiac surgical cohorts or critically ill patients in the ICU. A cross-sectional study exhibited that SBP variability was independently associated with AKI in patients undergoing aortic dissection surgery, while DBP variability demonstrated no significant link to AKI following aortic dissection surgery.^13^ Another prospective observational analysis elucidated that 24-hour ARV-SBP was significantly higher in ICU patients who developed AKI; the incidence of AKI increased from 14.0% (ARV ≤ 6 mmHg) to 73.9% (ARV > 14 mmHg).^14^ Our findings further suggest that the association between ARV-SBP and AKI may likewise be present among patients undergoing TAVR.

Notably, TAVR was primarily indicated for high-risk patients with aortic stenosis who are ineligible for conventional surgical aortic valve replacement. With an ageing population, the incidence of valvular degenerative disease was rising continuously.^15^ The mean age of 85 years among the included patients with TAVR was included in our baseline tables. The elderly population exhibits unique blood pressure changes, with a more prevalent isolated systolic hypertension defined as SBP ≥ 140 mmHg and DBP < 90 mmHg.^16^ Thus, SBP fluctuations might be more impactful in patients with TAVR, which became the reason why ARV-SBP was more closely associated with the AKI incidence than ARV-DBP. In addition, arterial stiffness, atherosclerosis, and autonomic dysfunction amplify BPV in older adults.^17^ Thus, the focus of clinical practice towards patients with TAVR was supposed to prioritize the changes of ARV-SBP, rather than merely concentrating on the control of absolute blood pressure levels.

The mechanism presenting a positive correlation between ARV-SBP and AKI following TAVR has not yet been elucidated. The intraoperative use of contrast medium and prolonged low perfusion pressure might be pivotal factors. Large volumes of contrast medium are routinely employed during TAVR for aortic root angiography, valve positioning, and deployment confirmation.^18^ Renal vasoconstriction and direct tubular toxicity are induced by contrast medium, thereby increasing AKI odds.^19^ Moreover, acute hypotension is frequently related to rapid ventricular pacing and valve deployment during TAVR.^20^ Renal perfusion pressure may influence sustained hypotension, which impairs renal blood flow and glomerular filtration rate (GFR), leading to ischaemic tubular injury.^21^ The clearance of contrast agents is also delayed by reduced GFR, prolonging retention time within the kidneys and further exacerbating nephrotoxicity.^22^ Haemodynamic instability and impaired vascular regulation are implied by postoperative elevations in ARV-SBP.^23^ Renal autoregulation ability is challenged by such BPV, rendering the patient’s renal perfusion in a state of repeated instability,^24^ which may be related to increased AKI odds.

Certain limitations were identified. First, data are primarily derived from a single-centre cohort of the US population, which limits generalizability to other countries or patients within the medical systems. Second, as an observational analysis, while the association between ARV-SBP and the incidence of AKI following TAVR is observed, the causal association has not yet been elucidated. Prospective or mechanism research is further required to explore. Third, while statistically adjusting for multiple potential covariates that may influence the outcome, inherent limitations of the MIMIC database mean that other unrecorded or unmeasured confounding factors (such as individual genetic background) may still exert potential effects on the study results. Finally, only patients with TAVR admitted to the ICU post-procedure are included; non-ICU patients with TAVR are ignored. The clinical characteristics, management strategies, and prognosis of this subset of patients may differ, such that the conclusions cannot be generalized to the entire population following TAVR. Future studies are warranted to further investigate the association between BPV and clinical outcomes in patients following TAVR who are not admitted to the ICU, conducting comparative analyses with data from ICU-admitted patients. This will enable a comprehensive evaluation of the link between blood pressure management and prognosis across groups with varying disease severity, thereby providing a more comprehensive evidence base for individualized perioperative management.

## CONCLUSION

In conclusion, higher ARV-SBP was associated with increased odds of AKI following TAVR. In clinical practice, alongside controlling the absolute SBP level, close attention should be paid to fluctuations in ARV-SBP, which may facilitate early risk identification of AKI after TAVR. Nevertheless, our results only support an observational association and cannot prove that stabilizing SBP variability reduces the odds of AKI. Prospective studies are needed in the future to further validate the value of ARV-SBP in risk stratification.

## Supplementary Material

ivag227_Supplementary_Data

## Data Availability

The data and materials in the current study are available from the corresponding author on reasonable request.
