# Supplementary material for: Association Between Blood Pressure Variability and Acute Kidney Injury Following Transcatheter Aortic Valve Replacement: A Study Based on the Medical Information Mart for Intensive Care-IV Database
Source: Interdiscip Cardiovasc Thorac Surg. 2026 Aug 14;41(11):ivag227. doi: 10.1093/icvts/ivag227 (PMC13730602; doi:10.1093/icvts/ivag227)
Supplement: Supplementary file 1 [file ivag227_supplementary_data.zip › Supplementary Figure Legends.docx]

**Supplementary Figure Legends**

Figure S1 Directed acyclic graph for the primary adjustment set.AKI, acute kidney injury; ARV, average real variability; DBP, diastolic blood pressure; KDIGO, Kidney Disease: Improving Global Outcomes; SBP, systolic blood pressure.
