# Supplementary material for: Association Between Blood Pressure Variability and Acute Kidney Injury Following Transcatheter Aortic Valve Replacement: A Study Based on the Medical Information Mart for Intensive Care-IV Database
Source: Interdiscip Cardiovasc Thorac Surg. 2026 Aug 14;41(11):ivag227. doi: 10.1093/icvts/ivag227 (PMC13730602; doi:10.1093/icvts/ivag227)
Supplement: Supplementary file 1 [file ivag227_supplementary_data.zip › Table S1-S7.docx]

**Table S1 ICD-9 and ICD-10 Codes for Identifying TAVR**

| **Surgery type** | **ICD codes** | **ICD version** |
| --- | --- | --- |
| TAVR | 3505 | 9 |
|  | 3506 | 9 |
|  | 02RF37H | 10 |
|  | 02RF37Z | 10 |
|  | 02RF38H | 10 |
|  | 02RF38Z | 10 |
|  | 02RF3JH | 10 |
|  | 02RF3JZ | 10 |
|  | 02RF3KH | 10 |
|  | 02RF3KZ | 10 |
|  | X2RF332 | 10 |
| TAVR, Transcatheter Aortic Valve Replacement; ICD, **International Classification of Diseases.** | | |

**Table S2. Early postoperative management characteristics during the ARV exposure window**

| **Characteristic** | **Overall (N=340)** | **No AKI (N=233)** | **AKI (N=107)** | **P value** |
| --- | --- | --- | --- | --- |
| Documented fluid input, mL | 3054 [2204, 4081] | 2903 [2120, 3818] | 3593 [2417, 5238] | <0.001 |
| Documented fluid output, mL | 1980 [1426, 2850] | 1900 [1375, 2784] | 2090 [1482, 2862] | 0.554 |
| Documented fluid balance, mL | 996 [16.8, 2063] | 872 [-40, 1795] | 1285 [278, 3676] | 0.002 |
| Documented diuretic use | 105 (30.9%) | 52 (22.3%) | 53 (49.5%) | <0.001 |
| Documented vasopressor use | 165 (48.5%) | 100 (42.9%) | 65 (60.7%) | 0.002 |
| Documented epinephrine use | 14 (4.1%) | 4 (1.7%) | 10 (9.3%) | 0.002 |
| Documented invasive mechanical ventilation | 117 (34.4%) | 62 (26.6%) | 55 (51.4%) | <0.001 |
| Values are presented as mean (SD), median [IQR], or n (%). Continuous variables were compared using Welch’s t-test when the absolute sample skewness was <1 and the Mann–Whitney U test otherwise. Categorical variables were compared using Pearson’s chi-square test or Fisher’s exact test when expected cell counts were <5. All P values are two-sided.。  These variables were measured concurrently with ARV exposure and may reflect disease severity or act as potential mediators; thus, they were not incorporated into Model M2.  AKI, Acute Kidney Injury; ARV, Average Real Variability. | | | | |

**Table S3 Covariate sensitivity analyses**

| **Model** | **Covariate change** | **ARV-SBP OR per 5 mmHg (95% CI)** | **P value** | **EPV** | **Maximum**  **VIF** | **Converged** |
| --- | --- | --- | --- | --- | --- | --- |
| M2 | Primary adjustment set | 1.34 (1.03–1.74) | 0.029 | 17.8 | 1.10 | Yes |
| M3 expanded baseline | M2 + hypertension, prior myocardial infarction, atrial fibrillation/flutter, and chronic pulmonary disease | 1.35 (1.04–1.76) | 0.027 | 10.7 | 1.14 | Yes |
| eGFR substitution | Replace creatinine with eGFR | 1.40 (1.07–1.83) | 0.014 | 17.8 | 1.08 | Yes |
| Charlson substitution | Replace diabetes and heart failure with Charlson index | 1.36 (1.05–1.77) | 0.021 | 21.4 | 1.16 | Yes |
| M2: adjustments for age, sex, median serum creatinine measured within 7 days prior to the recorded procedural date, diabetes mellitus, and congestive heart failure;  ARV-SBP, Average Real Variability of Systolic Blood Pressur; eGFR, eGFR, Estimated Glomerular Filtration Rate; EPV, Events Per Variable; VIF, Variance Inflation Factor. | | | | | | |

**Table S4. Primary model sequence for dynamic creatinine-based KDIGO AKI**

| **Model** | **ARV-SBP OR**  **per 5 mmHg (95% CI)** | **ARV-SBP P value** | **ARV-DBP OR**  **per 5 mmHg (95% CI)** | **ARV-DBP P value** |
| --- | --- | --- | --- | --- |
| M0 | 1.30 (1.02–1.67) | 0.038 | 1.13 (0.79–1.61) | 0.518 |
| M1 | 1.29 (1.01–1.66) | 0.045 | 1.12 (0.78–1.60) | 0.552 |
| M2 | 1.34 (1.03–1.74) | 0.029 | 1.12 (0.77–1.62) | 0.559 |
| Note: All odds ratios (ORs) represent the effect estimates per 5 mmHg increment in ARV. All models utilized the identical full study sample (N = 340, AKI events = 107). M0: without adjustments on covariates; M1: adjustments for age and sex; M2: adjustments for age, sex, median serum creatinine measured within 7 days prior to the recorded procedural date, diabetes mellitus, and congestive heart failure.  AKI, Acute Kidney Injury; KDIGO, Kidney Disease: Improving Global Outcomes; ARV-SBP, Average Real Variability of Systolic Blood Pressur; ARV-DBP, Average Real Variability of Diastolic Blood Pressure. | | | | |

**Table S5. BP measurement sensitivity analyses**

| **ARV specification** | **N/events** | **ARV-SBP OR (95% CI)** | **ARV-SBP**  **P value** | **ARV-DBP OR (95% CI)** | **ARV-DBP**  **P value** |
| --- | --- | --- | --- | --- | --- |
| Raw temporal sequence | 340/107 | 1.34 (1.03–1.74) | 0.029 | 1.12 (0.77–1.62) | 0.559 |
| Hourly aggregated | 338/106 | 1.38 (1.02–1.87) | 0.037 | 0.81 (0.52–1.26) | 0.354 |
| Invasive only | 294/89 | 1.57 (1.16–2.13) | 0.004 | 0.79 (0.39–1.61) | 0.519 |
| Non-invasive only (Firth) | 76/25 | 1.16 (0.65–2.06) | 0.617 | 1.30 (0.77–2.21) | 0.331 |
| Raw + corresponding mean BP adjustment | 340/107 | 1.50 (1.14–1.98) | 0.004 | 1.36 (0.91–2.05) | 0.138 |
| All odds ratios (ORs) represent the effect estimates per 5 mmHg increment in ARV. All rows were fitted with the M2 covariate adjustment set. Firth penalized logistic regression was only applied to analyses restricted to noninvasive blood pressure. Rows labeled “BP adjustment” included the corresponding 24-hour mean SBP or mean DBP as an additional covariate on top of Model M2.  M2: adjustments for age, sex, median serum creatinine measured within 7 days prior to the recorded procedural date, diabetes mellitus, and congestive heart failure.  ARV-SBP, Average Real Variability of Systolic Blood Pressur; ARV-DBP, Average Real Variability of Diastolic Blood Pressure; BP, Blood Pressure.  ① Hourly-aggregated sensitivity analysis: the initial 24-hour ICU window was split into consecutive full-hour intervals starting from ICU admission time, and median SBP and DBP values were calculated for each valid hour. Blood pressure differences were only computed between two sequentially numbered hourly segments. This analysis required a minimum of 6 valid hours generating at least 5 paired inter-hour differences. ② Isolated invasive-only and noninvasive-only ARV analyses followed the primary analysis thresholds: ≥6 valid paired recordings and a minimum 12-hour monitoring time span. ③ Additional adjustment for corresponding 24-hour mean blood pressure on top of M2: 24-h mean SBP and mean DBP were defined as the arithmetic mean of all quality-controlled eligible blood pressure pairs within the same exposure window, with the same priority rule for invasive blood pressure retained as in the primary analysis | | | | | |

**Table S6. AKI-definition sensitivity analyses**

| **Outcome group** | **Outcome definition** | **N/events** | **ARV-SBP OR per 5 mmHg (95% CI)** | **P value** |
| --- | --- | --- | --- | --- |
| Fixed-reference creatinine outcomes | ①Lowest creatinine in previous 24 h | 311/66 | 1.04 (0.76–1.42) | 0.814 |
|  | ②Most recent creatinine in previous 24 h | 311/65 | 1.05 (0.76–1.43) | 0.784 |
|  | ③Median creatinine in previous 7 d | 340/74 | 1.12 (0.84–1.49) | 0.455 |
|  | ④First creatinine in current hospitalization | 251/50 | 1.14 (0.80–1.63) | 0.467 |
|  | ⑤Historical median, 365–7 d before procedure date | 331/68 | 0.91 (0.67–1.25) | 0.574 |
| KDIGO severity/combined outcomes | Dynamic creatinine KDIGO Stage 1–3 | 340/107 | 1.34 (1.03–1.74) | 0.029 |
|  | Dynamic creatinine KDIGO Stage 2–3 (Firth) | 340/21 | 1.16 (0.73–1.83) | 0.537 |
|  | Combined creatinine/urine-output/CRRT KDIGO | 340/129 | 1.39 (1.08–1.80) | 0.011 |
| All effect estimates were derived from Model M2, with ORs corresponding to each 5 mmHg increment in ARV-SBP. Firth penalized logistic regression was utilized for analyses of creatinine-only KDIGO Stage 2–3 AKI. Among the fixed reference creatinine definitions, the criterion of serum creatinine elevation ≥0.3 mg/dL was only applied to creatinine measurements obtained within the first 48 hours after ICU admission.  M2: adjustments for age, sex, median serum creatinine measured within 7 days prior to the recorded procedural date, diabetes mellitus, and congestive heart failure.  ARV-SBP, Average Real Variability of Systolic Blood Pressur; AKI, Acute Kidney Injury; KDIGO, Kidney Disease: Improving Global Outcomes.  ① Lowest creatinine within 24 hours before the index procedure date; ② Creatinine measurement closest to the index procedure date within the preceding 24 hours; ③ Median creatinine measured over the 7 days before the index procedure date; ④ First inpatient creatinine measurement obtained prior to the index procedure date during the current hospitalization; ⑤ Historical median creatinine measured 365 to 7 days before the index procedure date. For all above fixed reference definitions, the absolute creatinine rise criterion (≥0.3 mg/dL) was only applied to creatinine assays collected within the first 48 hours post ICU admission, whereas the fold-increase criteria were used for all evaluable measurements throughout the 7-day post-admission window. | | | | |

**Table S7. KDIGO stage distribution**

| **Stage** | **Creatinine-based KDIGO, n (%)** | **Combined KDIGO, n (%)** |
| --- | --- | --- |
| 0 | 251 (69.0%) | 228 (62.6%) |
| 1 | 92 (25.3%) | 115 (31.6%) |
| 2 | 11 (3.0%) | 9 (2.5%) |
| 3 | 10 (2.7%) | 12 (3.3%) |
| Note: Separate evaluable risk sets of 364 patients were used for creatinine-based KDIGO and combined KDIGO definitions, respectively. The combined KDIGO stage was defined as the maximum stage derived from creatinine, urine output and CRRT criteria at each evaluable time point, with the peak stage identified within the first 7 days after ICU admission. | | |
